# A Case of Japanese Encephalitis with a Fatal Outcome in an Australian Who Traveled from Bali in 2019

**DOI:** 10.3390/tropicalmed5030133

**Published:** 2020-08-19

**Authors:** Alyssa T. Pyke, Keat Choong, Frederick Moore, Sanmarié Schlebusch, Carmel Taylor, Glen Hewitson, Jamie McMahon, Neelima Nair, Peter Moore, Mitchell Finger, Peter Burtonclay, Sarah Wheatley

**Affiliations:** 1 Public Health Virology Laboratory, Forensic and Scientific Services, Coopers Plains, QLD 4108, Australia; Frederick.Moore@health.qld.gov.au (F.M.); Sanmarie.Schlebusch@health.qld.gov.au (S.S.); Carmel.Taylor@health.qld.gov.au (C.T.); Glen.Hewitson@health.qld.gov.au (G.H.); Jamie.McMahon@health.qld.gov.au (J.M.); Neelima.Nair@health.qld.gov.au (N.N.); Peter.Moore2@health.qld.gov.au (P.M.); Mitchell.Finger@health.qld.gov.au (M.F.); Peter.Burtonclay@health.qld.gov.au (P.B.); Sarah.Wheatley@health.qld.gov.au (S.W.); 2 Sunshine Coast University Hospital, Birtinya, QLD 4575, Australia; Keat.Choong@health.qld.gov.au; 3 School of Medicine, The University of Queensland, St Lucia, QLD 4072, Australia

**Keywords:** Japanese encephalitis, Japanese encephalitis virus, flavivirus, Bali, Australia, CSF, whole genome sequencing

## Abstract

A severe case of Japanese encephalitis virus (JEV) infection, resulting in fatality, occurred in an unvaccinated Australian male traveler from Bali, Indonesia, in 2019. During hospitalisation in Australia, patient cerebrospinal fluid (CSF) yielded JEV-specific IgM antibodies and RNA, and an isolate of the virus. Ongoing transmission of JEV in Bali underscores this pathogen as a public health risk and the importance of appropriate health, vaccination and mosquito avoidance advice to prospective travelers to the region.

## 1. Patient Case History and Clinical Presentation

An unvaccinated, 59-year-old male, who was previously fit and well and routinely spent extended stays with an average duration of three months in Bali, traveled from Bali to his home in Queensland, Australia in early November, 2019. Six days after his departure from Bali, he became symptomatic and on day three of his illness, he presented at a Queensland hospital emergency department with fatigue, fever, headache and intermittent confusion. Although he experienced mosquito bites during his most recent stay in Bali, the patient had not been bitten by ticks or animals, did not have a rash and was not demonstrating arthralgia, myalgia, cardiorespiratory, gastrointestinal or urinary symptoms. The patient was kept in hospital overnight for observation and by the following morning, his condition had substantially deteriorated with the onset of flaccid paralysis and increased neurological dysfunction. He was moved to the intensive care unit (ICU) for intubation and ventilation. 

## 2. Hospital Examination and Laboratory Findings

**Figure 1:**
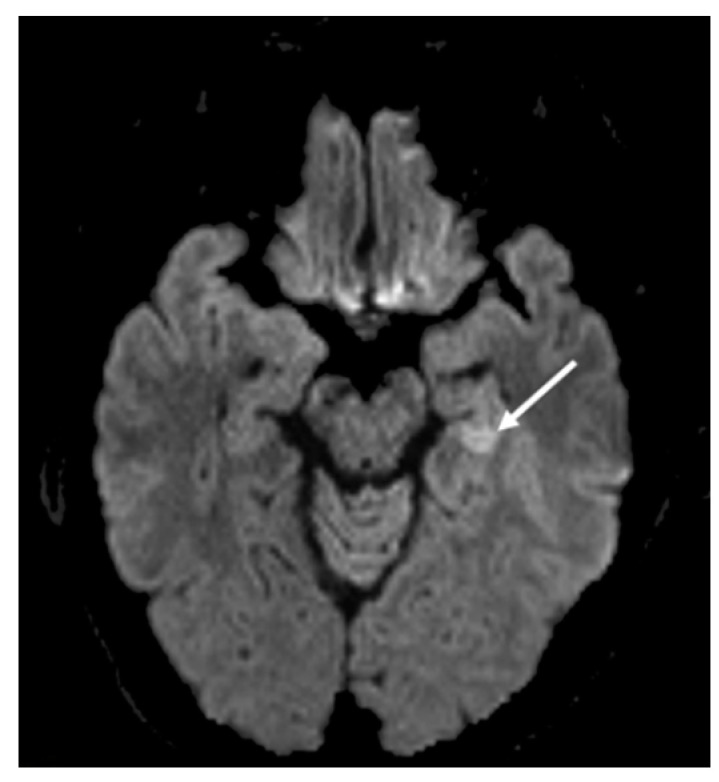
Fluid-attenuated inversion recovery (FLAIR), magnetic resonance imaging (MRI) sequence demonstrating hyperintensity and restricted diffusion in the left mesial temporal lobe (white arrow).

On day four of his illness and 24 h after hospital admittance, the patient’s symptoms of fever and disorientation were exacerbated, and he developed flaccid paralysis. He was moved to the intensive care unit (ICU) and required intubation and ventilation due to a decreased level of consciousness. A lumbar puncture was performed and the cerebrospinal fluid (CSF) sample collected demonstrated pleocytosis with 98% mononuclear cells, but normal glucose and protein parameters. The patient was empirically treated with ceftriaxone, ampicillin and acyclovir. Magnetic resonance imaging (MRI) revealed fluid-attenuated inversion recovery (FLAIR) hyperintensity and restricted diffusion in the left mesial temporal lobe (Figure 1). An electroencephalogram (EEG) further indicated diffuse slowing of background rhythms indicative of encephalopathy, but no epileptiform features.

The CSF collected on day four of illness was submitted for microbiological investigations. Bacterial culture yielded no growth and cryptococcus antigen testing was negative. Herpes simplex virus (HSV) RNA was not detected by reverse transcription real-time polymerase chain reaction (RT-rPCR). 

**Figure 2:**
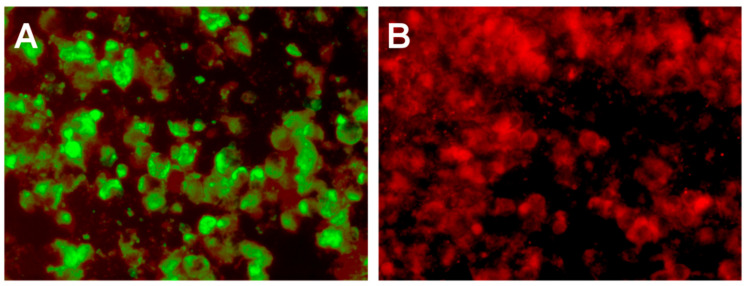
In vitro culture of Japanese encephalitis virus (JEV) from the cerebrospinal fluid (CSF) sample in *Aedes albopictus* C6/36 cells. Cell culture monolayers were analysed by immunofluorescent antibody assay (IFA) [4] using an anti-JEV monoclonal antibody, 6B4A-10 (Catalogue number MAB8743, Merck, Sigma-Aldrich, Australia). (**A**) CSF-inoculated, IFA-positive (green stain), JEV-infected C6/36 cells and (**B**) uninfected, IFA-negative C6/36 cells.

Alternate viral aetiologies were considered, and the CSF was further analysed to exclude a flavivirus infection [1]. Specific anti-Japanese encephalitis virus (JEV) IgM antibodies were detected using a multiplexed, flavivirus microsphere immunoassay (FL-MIA) [2] and JEV RNA was detected by RT-rPCR [3]. The CSF sample also yielded a JEV isolate following one passage in *Aedes albopictus* mosquito C6/36 cells (Figure 2).

Specific patient anti-JEV IgM antibodies were also detected by FL-MIA in a serum sample collected on day six after onset of symptoms, and an anti-flavivirus IgG antibody seroconversion determined via an in-house-developed enzyme-linked immunosorbent assay (ELISA) [4] was confirmed in a serum sample collected on day 12 of his illness.

## 3. Whole Genome Sequencing and Phylogenetic Analysis

The patient’s JEV isolate (JEV Bali 2019) was further characterised by massive parallel sequencing using the Nextera XT kit for cDNA library construction and paired-end (2 × 150 nucleotides) sequencing using the V2 mid-output kit on a NextSeq 500 machine (Illumina, San Diego, CA) as previously described [5]. A total of 16,585,742 raw sequencing reads were obtained and were processed using Geneious R10 version 10.2.6 software [6]. Complete genome assembly was achieved by mapping to a reference JEV genome (strain JEV/sw/Bali/93/2017 (JEV Bali 2017), GenBank accession number LC461961.1). A total of 10,601,804 reads with an average coverage depth of 103,358× (10,970 nucleotides) were mapped to JEV Bali 2017. 

**Figure 3:**
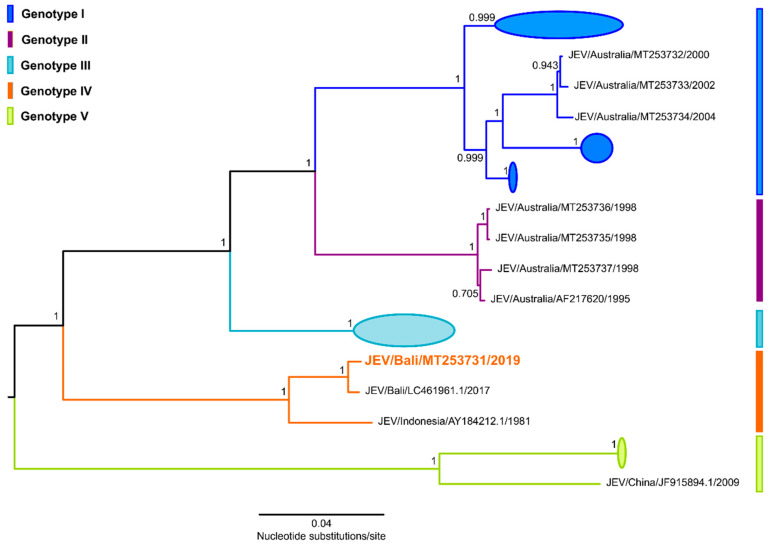
Midpoint rooted, approximately maximum likelihood phylogenetic tree estimated from 243 JEV complete coding region sequences (10,303 nucleotides). Tree construction was performed as previously described [5] using FastTree version 2.1.11 software and the general time reversible (GTR) nucleotide substitution model. Multiple sequence alignments were performed using the Multiple Alignment Using Fast Fourier Transform (MAFFT) program, version 7.450, and Geneious software, version 10.2.6. The five JEV genotypes are shown with major clades collapsed to highlight key JEV strains. Shimodaira–Hasegawa-like local support values are shown for selected nodes. The JEV Bali 2019 strain and corresponding designation within JEV genotype IV is shown in orange.

Phylogenetic analysis comparing the complete coding region (10,302 nucleotides) of JEV Bali 2019 with 242 other JEV strains (Figure 3) was performed using FastTree software and the general time-reversible nucleotide substitution model as previously described [5]. JEV Bali 2019 (GenBank accession number MT253731.1) grouped within JEV genotype IV and was most closely related to the strain JEV/sw/Bali/93/2017 isolated in Bali, Indonesia, 2017 (GenBank accession number LC461961.1) and JEV strain JKT6468 isolated in Indonesia, 1981 (GenBank accession number AY184212.1), sharing 99.23% and 95.38% nucleotide identity respectively, over the complete genome (10,970 nucleotides). Interestingly, JEV Bali 2019 was also shown to be distinctly different to the JEV isolates obtained during local outbreaks [7,8,9] and surveillance efforts [10,11] in nearby Australia, which belong to JEV genotypes I and II.

## 4. Patient Outcome

Despite some neurological improvement, where the patient regained consciousness and was able to communicate, he remained profoundly weak, ventilator-dependent and required a tracheostomy. His course was complicated by ventilator-associated pneumonia. After spending more than three months in ICU, there was no improvement in his ventilator state and the patient died after the decision was made to withdraw care. 

## 5. Discussion

Transmission of the flavivirus JEV to humans is incidental and occurs following the bite of an infected mosquito, particularly those belonging to the *Culex* genus. Whilst human infections are principally asymptomatic, the risk of exposure can increase where efficient amplifying hosts, such as domestic pigs and/or wading birds, are proximal to humans [7,12]. 

Japanese encephalitis (JE) remains a serious and incurable public health risk throughout Asia and the Pacific rim. This neurological syndrome is grossly underreported, and fewer than 1% of the estimated 68,000 human clinical cases occurring annually [13,14], are actually reported in endemic countries. JE predominantly affects children, meaning they constitute many of the 20–30% of JE cases with fatal outcome or they are among the 30–50% of survivors who develop irreversible neurologic or psychiatric sequelae. However, all age groups are vulnerable and risk of JEV infection increases in the absence of naturally-acquired or vaccine-induced immunity [13].

The overall risk of JE for people traveling to Asia from non-endemic regions is considered extremely low and less than one case of JE is estimated to occur per one million travelers [12]. However, risk of exposure can increase in unvaccinated travelers who have prolonged or repeated stays in endemic countries, and undertake activities in locations, and at the time of day, when mosquitoes are active [12]. 

Relevant to the case we describe, JEV transmission in Indonesia is widespread and is now endemic in 94% of the country’s provinces, including the popular tourist island of Bali [15,16]. JEV infections in travelers to Bali from non-endemic countries like Australia have been previously reported [17,18,19]. Safe vaccines are available against JEV [12], however, unvaccinated travelers continue to contract JE with serious consequences [19]. The unvaccinated JE case with fatal outcome we report had a repeated history of spending on average three months in Bali followed by three months in Australia. His prolonged stays in Bali, from where he subsequently contracted a JEV infection, highlight the fundamental necessity of appropriate medical, vaccination and mosquito avoidance advice to prospective travelers, and prioritisation of improved surveillance, diagnostic consideration and detection strategies for JEV and other transmissible pathogens.
